# Outpatient Testing for HIV in Italy, 2018–2023—Preliminary Data

**DOI:** 10.3390/microorganisms13030655

**Published:** 2025-03-13

**Authors:** Claudio Galli, Vincenza Regine, Anna Caraglia, Francesca Centrone, Maria Chironna, Gianluca Cruschelli, Massimo Farinella, Valentina Annachiara Orlando, Chiara Pasqualini, Monia Puglia, Lucia Pugliese, Laura Rancilio, Lara Tavoschi, Fabio Voller, Barbara Suligoi

**Affiliations:** 1 Independent Researcher, 00139 Rome, Italy; 2 AIDS Operations Center, Department of Infectious Diseases, Italian National Institute of Health, 00161 Rome, Italy; vincenza.regine@iss.it (V.R.); lucia.pugliese@iss.it (L.P.); barbara.suligoi@iss.it (B.S.); 3 Former General Directorate of Health Prevention, Ministry of Health, 00144 Rome, Italy; a.caraglia@sanita.it; 4 Hygiene Operational Unit, Policlinico Consorziale di Bari, 70124 Bari, Italy; francesca.centrone@policlinico.ba.it; 5 Interdisciplinary Department of Medicine, University of Bari, 70124 Bari, Italy; maria.chironna@uniba.it (M.C.); valentina.orlando@uniba.it (V.A.O.); 6 Department of Translational Research and New Technologies in Medicine and Surgery, University of Pisa, 56124 Pisa, Italy; g.cruschelli@studenti.unipi.it (G.C.); lara.tavoschi@unipi.it (L.T.); 7 Mario Mieli, LGBTQIA+ Culture Center, 00146 Rome, Italy; massimofarinella@gmail.com; 8 Piedmont Regional Service for the Epidemiology of Infectious Diseases (SeREMI), 15121 Alessandria, Italy; cpasqualini@aslal.it; 9 Research and Innovation Department (DAIRI), “SS Antonio e Biagio e C. Arrigo” University Hospital, 15121 Alessandria, Italy; 10 Tuscany Regional Health Agency (Agenzia Regionale di Sanità, ARS), 50141 Florence, Italy; monia.puglia@ars.toscana.it (M.P.); fabio.voller@ars.toscana.it (F.V.); 11 Caritas Ambrosiana, 20122 Milan, Italy; l.rancilio@caritasambrosiana.it

**Keywords:** HIV diagnosis, HIV testing, HIV epidemiology, diagnostic algorithms, population screening, supplemental tests

## Abstract

HIV testing is crucial towards the control of the Acquired Immune Deficiency Syndrome (AIDS) epidemic. Monitoring trends of human immunodeficiency virus (HIV) testing over time may help interpret the incidence of new HIV diagnoses and effectiveness of HIV testing strategies. We started a research project aimed at assessing testing rates for HIV infection among Italian outpatients in 2018–2023. Numeric data for screening, confirmatory, and monitoring tests obtained by a national register were compared with the numbers of adult residents, newly diagnosed HIV infections, and patients undergoing treatment. The number of screening tests declined from 1,133,377 in 2018 to 889,972 in 2020 and increased to 1,096,822 in 2023. HIV-RNA tests showed a similar pattern, whereas confirmatory immunoblots did not vary significantly over time. The ratio of screening tests to adult residents was higher in North-West (2.87%) and North-East (2.31%) Italy compared to South Italy and the islands (1.47%), indicating that screening should be enhanced in the latter area. We observed differences between the ratio of screening tests and the incidence of newly diagnosed HIV infections by geographic area. Discrepancies in the number of screening and confirmatory tests needed for each new diagnosis suggest repeated testing on people already diagnosed and possible data reporting issues. The monitoring of HIV screening tests at the national and regional levels can provide essential data to interpret trends in HIV epidemiology and plan relevant testing strategies over time.

## 1. Introduction

A global commitment to end the Acquired Immune Deficiency Syndrome (AIDS) epidemic as a public health threat by 2030 has been established by UNAIDS [1]. To achieve this goal, countries were urged to adopt the UNAIDS Fast-Track strategy, which included three interim targets defined as “the continuum of care” (or treatment cascade) that classifies individuals with Human Immunodeficiency Virus (HIV) infection who are diagnosed, linked to care, retained in care, on antiretroviral therapy, and virally suppressed, with the aim of assessing the effectiveness of health care and treatment from a population-level health perspective. This model proposed three targets—90% of people with HIV infection to be diagnosed, 90% of those diagnosed to receive antiviral treatment, and 90% of treated patients to achieve a status of viral suppression—that should have been reached by 2020. Through combined political, social, and economic efforts, substantial progress was made, but nevertheless only a few countries met these targets by 2020. Thus, in 2021, a new global strategy with a focus on inequalities was proposed by UNAIDS with the aim of reducing new HIV infections from 1.65 million to less than 370,000 per year and AIDS-related deaths from 718,000 to less than 250,000 per year [2,3]. The three interim targets from the 2015 strategy were raised to 95% diagnosed, 95% treated, and 95% suppressed. The first step is to achieve the ‘95% diagnosed’ goal. To reduce the proportion of undiagnosed individuals, promotion of HIV testing and facilitated access to testing should be emphasized, especially among high-risk populations. In this context, some elements that can reduce barriers to HIV testing should be reinforced, such as normalizing HIV testing (including opt-out testing strategies) and incorporating it into routine care, reducing stigma and discrimination, stressing the benefits of early diagnosis and treatment, and increasing the availability of self-tests, rapid tests, and community-based testing, possibly at no cost. In fact, free HIV testing and treatment in many countries across the world has already saved millions of lives and is helping to reduce the numbers of new HIV infections. However, despite all the remarkable gains made over the past two decades, testing and treatment services are still missing millions of people, many of whom belong to marginalized key populations.

In Italy, the estimates of the continuum of care show that 94% of people living with HIV are diagnosed (4), equal to an approximate number of 9000 individuals unaware of their serostatus. Surveillance data indicate that in 2023, 60.1% of newly diagnosed HIV infections are diagnosed late, a proportion significantly higher than the 52.7% reported in Europe. Moreover, the proportion of late presenters in Italy has progressively increased in the last 12 years. Most of them are older than 30 years and heterosexual [4], suggesting that the perception of HIV risk is low in the general population and that at-risk behaviors are hardly recognized. Although HIV testing in public services in Italy is anonymous and free, HIV testing is still lagging. In the last decade, the incidence of new HIV diagnoses (number of individuals who receive the first HIV positive test result by 100,000 residents) in Italy has decreased from 6.4 in 2014 to 4.0 in 2023. However, the lack of a systematic collection of data on HIV tests performed at a national level makes it difficult to discriminate whether this trend is attributable to a reduction in new HIV infections or to a decrease in the number of people being tested for HIV and consequently in the number of people discovered as HIV positive.

In this view, monitoring trends of HIV testing over time may help interpret the incidence trends of new HIV diagnoses and allow for some evaluation of the effectiveness of HIV testing strategies. We therefore started a research project, financed by the Italian Ministry of Health, aimed at analyzing official reports on HIV testing in outpatients to better understand the overall picture of HIV testing in Italy and highlight potential gaps.

## 2. Materials and Methods

National data on HIV testing were obtained from the Italian Ministry of Health (MoH), which collects on a yearly basis essential data on diagnostic tests carried out on outpatients accessing public health services in Italy.

Public laboratories located in hospitals, local health services, and universities, as well as other laboratories affiliated with the National Health System, collect information on the number and type of tests performed. These data are sent to the regional reference center and then to the General Directorate for Informatic Systems of the MoH. Those data are fully anonymous, and no information on age and gender, nor clinical or immunological data, is available.

The national database centralized in the MoH is not publicly available but may be accessed upon request either for institutional reasons or for research projects, such as ours. We obtained data on HIV testing from all 21 regions and autonomous provinces for the years 2018–2023. Every test is coded according to the codes reported in the 2022 version of the National Health Care Range of Fees (Italian name: Nomenclatore tariffario).

We analyzed codes that identify first-line tests for HIV screening (defined as “screening tests” from now on) and those that identify second-line tests employed to confirm first-line test reactivity (defined as “confirmatory tests” from now on). Other HIV tests were grouped together and include immunoassays that detect HIV-1 p24 antigen, viral culture, and tests that measure antiviral drug resistance.

**Figure 1:**
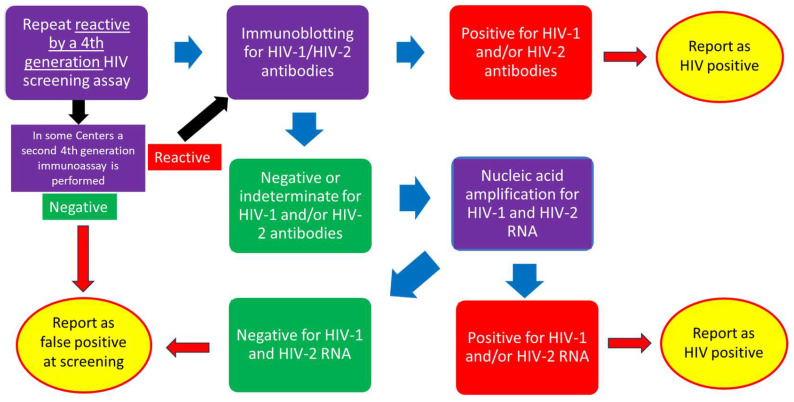
Schematic workflow of the HIV diagnostic algorithm most frequently adopted in Italy. Starting from reactivity by any screening test (top left), the large blue arrows indicate a sequential testing approach (supplemental testing by HIV-specific immunoblots and nucleic acid amplification methods, if needed), and the red arrows indicate the final step according to the results of supplemental testing. The black arrows indicate an alternative first step for confirmation (second screening test) adopted in some areas.

According to the indications issued by the Italian Society of Clinical Microbiology (AMCLI) [5], HIV screening tests currently used in Italy are 4th generation immunoassays that detect simultaneously HIV-1 and HIV-2 specific antibodies and the HIV-1 p24 antigen, whereas confirmatory tests are either immunoblots able to detect and differentiate antibodies towards HIV-1 and HIV-2 antigens or nucleic acid amplification tests (NAT) that detect HIV nucleic acid (HIV-RNA). Figure 1 describes the confirmatory pathway most frequently used in Italy. Samples that are reactive by a screening test are usually processed by immunoblot, and, if positive, an HIV-positive report is issued. In some centers, a second screening test is performed to increase specificity and reduce the number of samples to be analyzed by immunoblots. When immunoblots yield a negative or indeterminate result, a further step (testing for HIV-RNA by NAT) is needed.

The denominators used to describe rates are (a) the number of adult residents (≥18 years old) by region or autonomous province and year, available from the Central Institute of Statistics (ISTAT) [6]; (b) the number of newly diagnosed HIV infections (NHD), by year [4], reported to the Italian Surveillance system of new HIV diagnoses [7]; and (c) the number of people with HIV currently receiving antiretroviral treatment (ART), derived from estimates of the continuum of care in Italy [8].

We calculated the HIV screening rate in adult residents (ratio between the number of screening tests and the number of adult residents), the average number of screening tests per NHD (ratio between the number of screening tests and the number of NHD), the average number of confirmatory immunoblot tests per NHD (ratio between the number of immunoblot tests and the number of NHD), and the average number of HIV-RNA tests per person living with HIV receiving ART (ratio between the number of HIV-RNA tests and the number of people living with HIV receiving ART). These rates were disaggregated by year of testing (2018–2023), and geographical area in Italy [North-West (four regions), North-East (three regions and two autonomous provinces), Central (four regions), South and islands (eight regions)].

To assess temporal differences in the above-mentioned rates, the study period was divided into three periods of time: pre-COVID-19 2018–2019, COVID-19 2020–2021, and post-COVID-19 2022–2023. The differences were evaluated using the chi-square test and considered significant if the p-value was less than 0.05.

To assess the relationship between screening rates and NHD incidence, Pearson’s correlation coefficient was calculated by year of testing and diagnosis.

## 3. Results

### 3.1. Overview on HIV Tests

The number and distribution of HIV tests performed in outpatients accessing public health services in Italy are reported in Table 1. Screening tests are employed both in population screening and in the diagnostic process [5,9], representing the majority of HIV tests—84.0% of all tests performed, ranging between 82.9% and 85.1% across years. Confirmatory HIV immunoblots, widely employed as a second diagnostic step to confirm reactivity by first-level tests, represent 0.5% of all HIV reported tests and 0.6% of screening tests. The second most frequently reported test is HIV-RNA NAT, which represents 13.6% of all HIV tests; this test is mostly used to monitor people living with HIV and on treatment, whereas only a small fraction is attributable to screening confirmation.

**Figure 2:**
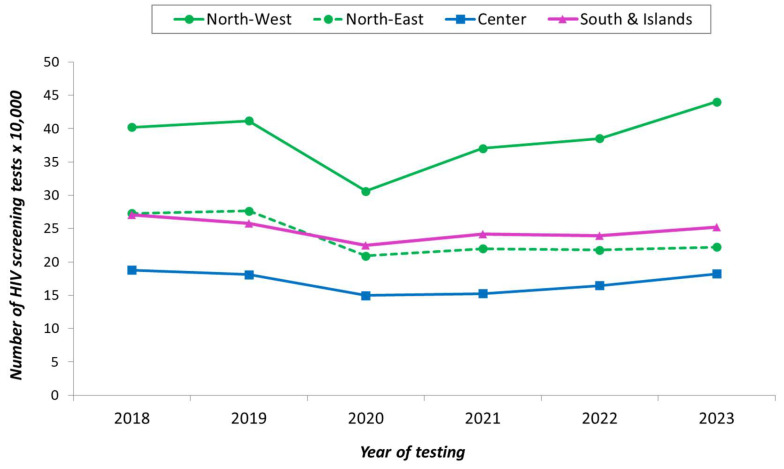
Number of HIV screening tests by geographical area and year of testing, Italy 2018–2023.

Other HIV tests represent less than 2% of all HIV tests performed among Italian outpatients. The temporal trend of the number of HIV screening tests by geographic area is shown in Figure 2.

### 3.2. HIV Testing Rates Among Adult Residents, New HIV Diagnoses, and Patients Receiving ART

**Figure 3:**
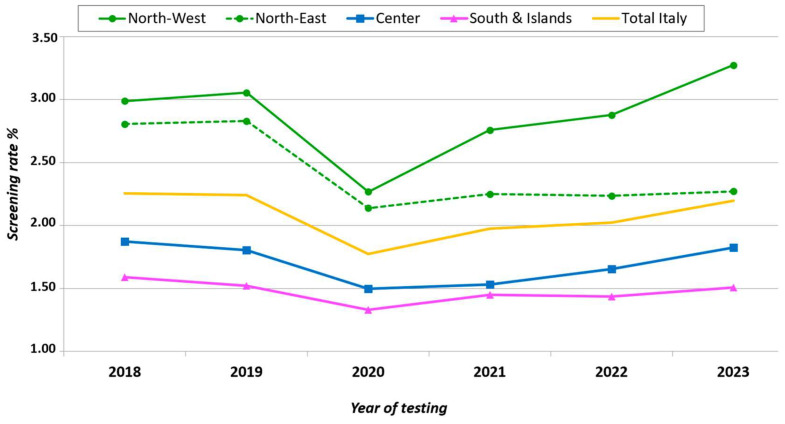
Rates of HIV screening per adult residents × 100 by area of residence, Italy, 2018–2023.

In the six years considered, the average HIV screening rate among adult residents was 2.06%, ranging between a maximum of 2.23% in 2018 and a minimum of 1.75% in 2020 (Figure 3). For the whole country, that rate was 2.26/100,000 adult residents in 2018, 2.24 in 2019, and 2.20 in 2023, almost back to the pre-pandemic years. We observed a different trend across the four geographical areas: in North-West Italy, the 2023 rate exceeded the pre-pandemic years, while in North-East Italy that rate remained low (2.27 in 2023 vs. 2.81 in 2018 and 2.82 in 2019), and in Central Italy and South Italy and the islands, the 2023 rates were almost the same compared to the pre-pandemic years.

**Figure 4:**
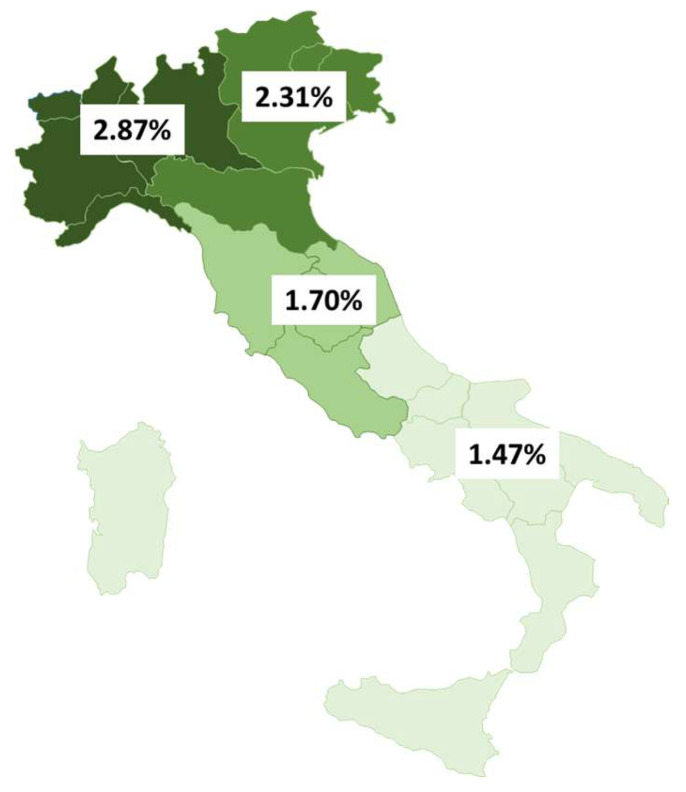
HIV screening rate in adult residents (number of screening tests/adult residents × 100) by geographical area, Italy, 2018–2023. North-West Italy: dark green; North-East Italy: green; Central Italy: light green; South Italy and the islands: pale green.

When disaggregated by four geographical areas, the HIV screening rate showed a decreasing gradient from North to South Italy across the years; overall, this rate ranged from 2.87% in North-West Italy to 1.47% in South Italy and the islands (Figure 4).

The average number of screening and confirmatory immunoblot tests per NHD and the average number of HIV RNA tests per patient receiving ART are shown in Table 2, by year. To mitigate the effect of COVID-19 on the reduced number of HIV screening tests observed in 2020, a stratified analysis of the entire study period into two-year periods (pre-COVID-19: 2018–2019, COVID-19 period: 2020–2021, post-COVID-19: 2022–2023) was performed. The average number of screening tests per NHD was 407 in 2018–2019, 554 in 2020–2021, and 469 in 2022–2023, with a statistically significant difference (*p* < 0.05) among groups. Similarly, the difference in the average number of confirmatory immunoblots per NHD was statistically significant (*p* < 0.05) among periods being, respectively, 2.1, 4.2, and 2.6.

**Figure 5:**
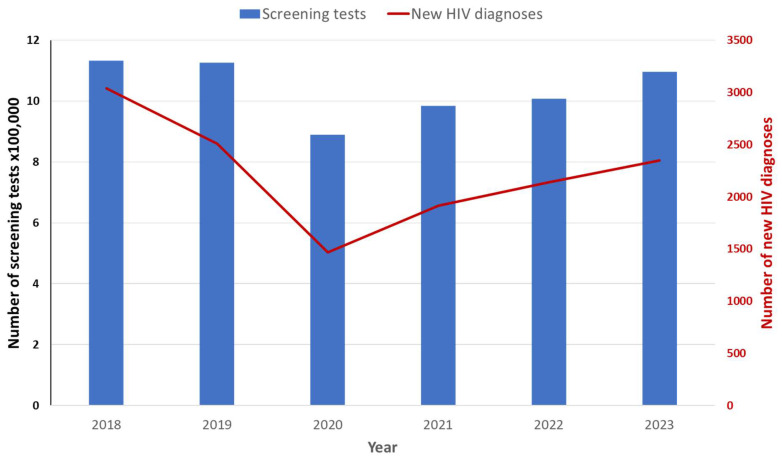
Temporal trend of the number of HIV screening tests (left Y axis) and the number of new HIV diagnoses (right Y axis); Italy, 2018–2023.

**Figure 6:**
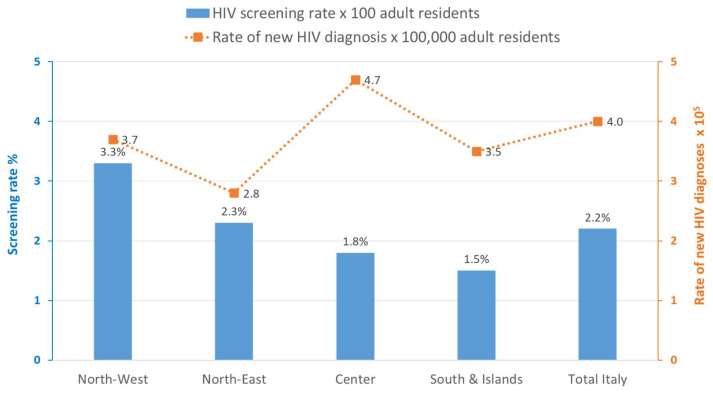
HIV screening rate × 100 adult residents (blue bars) and HIV incidence of NHD x 100,000 adult residents (dotted line) by geographic area, Italy, 2023.

The temporal trend of the number of screening tests and the number of NHD shows a similar pattern, with a decrease in 2020 and an increase in the following years (Figure 5). However, when comparing the trends of the HIV screening rate × 100 adult residents and the incidence of NHD by geographic area (Figure 6), no correlation (r2 = 0.0195) was observed either in 2023 or in the previous years.

## 4. Discussion

Exploring screening rates for HIV infection over time allows a better picture of the outcomes of awareness and enhanced screening campaigns aimed at reducing the number of undiagnosed cases and reinforcing health care policies focused on the new 95% diagnostic goal proposed by the UNAIDS in 2023 [2]. In Italy, the data from the national surveillance system for HIV reveal that the incidence of the infection is 4.0 per 100,000 residents, lower compared to that reported in Western European countries and the European Union (6.2 new diagnoses per 100,000), but more than 60% of cases are diagnosed late, with a number of CD4 lymphocytes lower than 350 cells/μL [4].

Up to now, a systematic collection of data on HIV tests performed at a national level in Italy is lacking. This is the first study that explores the number of HIV tests performed in Italy obtained from the research project PRONTI (translated from Italian: project on the number of outpatient HIV tests) aimed at analyzing the distribution of HIV tests among outpatients in Italy. Approximately one million adults per year were tested for HIV in the study period, with a relevant decrease in 2020 because of the restrictions due to the COVID-19 epidemic and limitations in accessing health services [10]; a similar decrease has been reported in other countries [11]. Our data show that the decrease in the number of screening tests observed in 2020 and 2021 was more relevant in North-West Italy, which was the area mostly affected by the COVID-19 epidemic [12]. HIV testing showed a rebound after 2020, although it did not fully return to pre-pandemic levels, especially in North-East Italy, which is puzzling since in that area test offering and testing conditions are quite similar to North-West Italy. In 2023, the percentage of tests per resident adult was 0.6%. This proportion is similar to that reported in 2023 in other Western European countries, showing a minimum in Greece (0.3%) and a maximum in France (11.0%) [13].

The low rate of confirmatory HIV immunoblot tests is in agreement with the low HIV incidence in Italy and the good specificity of screening tests currently used [9]. It is noted that the ratio of both screening and confirmatory immunoblot tests per NHD was significantly higher in the COVID-19 years compared to the pre- and post-COVID-19 period. In other terms, during the COVID-19 epidemic, more people were tested, but fewer new HIV cases were found. This may suggest that HIV testing in 2020 and 2021 was less focused on individuals at high risk and/or with HIV-indicative conditions, which represent the two most frequent reasons for undergoing HIV testing among NHD [4]. HIV testing may have been implemented in many individuals presenting with systemic symptoms that were probably caused by COVID-19 but could also be related to HIV, thus leading to a less targeted screening strategy for HIV.

Currently used HIV RNA NAT yields both a qualitative response (detection of HIV-RNA) and a quantitative evaluation (also called ‘viral load’, expressed in RNA copies/mL) and are mostly employed in monitoring people living with HIV, either treatment naïve or on ART. Only a small fraction is used to confirm an initial reactive screening test or to resolve results that are not classified by immunoblot, representing 10–15% of all immunoblot results [14,15]. Not surprisingly, the number of HIV RNA NAT showed a decrease in 2020 and an increase in the following years, most probably due to the cancelation/disruption of health services during the COVID-19 epidemic in Italy.

The annual rate of HIV screening tests per 100 adult residents shows a north-to-south decreasing gradient that remained quite constant throughout the study period. This may be ascribed to facilitated access to testing, more effective educational campaigns, a higher number of testing sites, or a greater awareness of at-risk exposure for HIV in Northern Italy. Interestingly, North-West Italy, which was heavily affected by the COVID-19 epidemic in 2020, showed the strongest rebound in HIV testing rate after 2020. Coding flaws and differences in the accuracy of regional reporting systems could also explain gaps between geographic areas.

The need for confirmatory testing on samples that are reactive by first-level tests employed for HIV screening or diagnosis stems from the low positive predictive value of any test, even if highly specific, in low-prevalence settings. Since the estimated HIV prevalence in Italy is about 0.23% [4,16], even with a 4th generation test with specificity over 99.8% [14,15], the positive predictive value for a reactive result would be around 50%. The approaches to HIV confirmation are different across countries and also within countries. Although the World Health Organization (WHO) suggested dismissing the employment of immunoblots due to high cost and delay in providing results [17], the employment of the newest versions of these tests that have a very quick turnaround time [18] has been recommended by the Centers for Disease Control (CDC) [19] and is still frequent in western countries. Another approach to HIV confirmation is to test samples reactive at an initial screening test by a second screening test [5] to reduce the number of immunoblots and/or HIV-RNA tests (Figure 1). Although this approach intends to reduce costs and shorten time to diagnosis, differences in sensitivity between the two screening tests in the early stage of infection have been reported [20] and may cause a false negative when the risk of transmission is higher [9]. This misclassification probability is reduced when samples that are negative by the second screening test are tested for HIV-RNA [20,21]. Appropriate national guidelines for an HIV testing algorithm would be useful in standardizing testing procedures.

In Italy, immunoblots (performed on samples reactive at the first or after the second screening test) are the preferred method to confirm HIV positivity. Therefore, we considered the number of these tests as a reliable proxy of samples reactive by a screening test. The ratio of immunoblots to screening tests was 0.6% overall and slightly higher (0.7–0.8%) in 2020 and 2021. Assuming a constant rate of false positives at screening of 0.2% (14, 15), an estimated proportion of 0.4% of all samples screened would be confirmed as HIV positive, and the absolute number of HIV positive samples detected over the six years considered would then be approximately 25,000. Nevertheless, this estimate is higher than the 13,421 new HIV diagnoses reported to the national surveillance system in 2018–2023 [4]. Some hypotheses can be made to explain this difference. First, several people may have been repeatedly tested: a region in North-West Italy estimated a 30% test repetition in the period 2018–2023 (average ratio of 1.30 between the number of screening tests and the number of individuals screened) [22]. Similarly, a study conducted in South Carolina (United States, US) over a 12-year period found a 34% repeated immunoblot testing among 12,504 HIV-infected individuals [23]. This finding suggests that some individuals probably want to be sure of the first positive test result, whereas others are retested when accessing a new health care service. Second, an unnecessary repetition of confirmatory immunoblot tests may have occurred, especially during the COVID-19 epidemic when several reports described the association between SARS-CoV-2 infection and false positive HIV screening test results [24]. This may also explain the divergent trend we found during the COVID-19 years between the decreased number of HIV screening tests as opposed to the increased number of immunoblot confirmatory tests. Third, we cannot rule out the underreporting of new HIV diagnoses to the national surveillance system.

Our paper has some limitations. First, we were able to investigate only HIV tests carried out in outpatients attending public health services, whereas we have no data on people tested in other settings such as hospitalized individuals, people tested in private health care services, in outreach and community settings, or people that used self-tests. Therefore, the total number of screening tests is to some extent underestimated. On the other hand, the ratio of screening tests to adult residents may be overestimated, as the same individual can undergo testing more than once. Furthermore, in some settings, confirmatory testing includes retesting samples that were reactive on the first screening test with a second screening method [5], thus increasing the number of screening tests. Finally, we cannot exclude local defaults in the data flow or faults in test coding that can lead to inaccuracies in data reporting.

## 5. Conclusions

Despite the reduction in HIV screening and monitoring tests during the COVID-19 years, HIV testing in Italy remained quite stable between 2018 and 2023 with an average screening rate of 0.6% among resident adults. A higher HIV screening rate was found in North and Central Italy, stressing the need to promote HIV testing in South Italy and the islands. Up to now, there are no national guidelines for an HIV testing algorithm that would be useful in standardizing testing procedures, shortening time to result, and optimizing costs. The monitoring of HIV screening tests at the national and regional levels can provide essential data to interpret trends in HIV epidemiology and plan relevant testing strategies over time [25].

## Figures and Tables

**Table 1:** Distribution of HIV diagnostic tests by type of test and year of testing, Italy, 2018–2023 ^1^.

Type of HIV Test	2018	2019	2020	2021	2022	2023	Total
All HIV tests N.	1,355,413	1,345,857	1,045,246	1,187,581	1,196,361	1,297,921	7,428,379
% of screening tests out of all HIV tests	83.6%	83.7%	85.1%	82.9%	84.2%	84.5%	84.0%
Screening rate/100 resident adults	0.5%	0.5%	0.7%	0.8%	0.5%	0.6%	0.6%
Confirmatory HIV immunoblot N.	5909	5931	6313	7755	5272	6417	37,597
% of confirmatory immunoblot out of HIV screening tests	0.5%	0.5%	0.7%	0.8%	0.5%	0.6%	0.6%
HIV-RNA NAT N.	199,021	193,908	127,306	160,122	157,877	168,824	1,007,058
% of HIV-RNA NAT out of all HIV tests	14.7%	14.4%	12.2%	13.5%	13.2%	13.0%	13.6%
Other HIV tests N.	17,106	19,072	21,655	34,994	25,681	25,858	144,366
% of all HIV tests	1.3%	1.4%	2.1%	2.9%	2.1%	2.0%	1.9%

^1^ N. = number; % = percentage; screening rate = ratio between the number of screening tests and the number of adult residents; confirmatory immunoblot = test that detects specific antibodies to different HIV-1 and HIV-2 antigens; HIV-RNA NAT = nucleic acid amplification test that detects HIV nucleic acid.

**Table 2:** Average number of HIV screening tests and confirmatory immunoblot tests per NHD, and HIV-RNA tests per patient receiving antiviral therapy (ART) by year, Italy, 2018–2023.

	2018	2019	2020	2021	2022	2023	Total
Number of new HIV diagnoses (NHD)	3038	2510	1470	1914	2140	2349	13,421
Average n. of screening tests per NHD	373	44	605	514	471	467	465
Average n. of confirmatory immunoblot tests per NHD	1.9	2.4	4.3	4.1	2.5	2.7	2.8
Number of patients receiving ART	121,000	123,000	125,000	127,000	130,000	131,000	757,000
Average n. of HIV RNA tests per patient receiving ART	1.6	1.6	1.0	1.3	1.2	1.3	1.3

## Data Availability

The dataset utilized in this article cannot be shared because of restrictions from the Italian Ministry of Health.

## Abbreviations

The following abbreviations are used in this manuscript:

AIDS	Acquired Immune Deficiency Syndrome
UN	United Nations
UNAIDS	United Nations Programme on HIV/AIDS
HIV	human immunodeficiency virus
GBD	Global Burden of Disease
MoH	Ministry of Health
AMCLI	Associazione Italiana Microbiologi Clinici
NAT	nucleic acid tests
ISTAT	Istituto Nazionale di Statistica
NHD	new HIV diagnosis
ART	antiretroviral therapy
WHO	World Health Organization
CDC	Centers for Disease Control
US	United States
